# Association between the use of weight management strategies and weight change among Australian adults over 12 months: an observational study

**DOI:** 10.1186/s12889-023-16277-4

**Published:** 2023-07-31

**Authors:** Ben Singh, Timothy Olds, Rachel Curtis, Ty Ferguson, Lisa Matricciani, Wendy J Brown, Dorothea Dumuid, Adrian Esterman, Carol Maher

**Affiliations:** 1 Alliance for Research in Exercise Nutrition and Activity (ARENA), University of South Australia, Adelaide, South Australia Australia; 2 University of Queensland, St Lucia, Brisbane, Australia

**Keywords:** Diet, Physical activity, Weight management, Weight loss, Obesity

## Abstract

**Background:**

Obesity is a growing, global public health issue. This study aimed to describe the weight management strategies used by a sample of Australian adults; examine the socio-demographic characteristics of using each strategy; and examine whether use of each strategy was associated with 12-month weight change.

**Methods:**

This observational study involved a community-based sample of 375 healthy adults (mean age: 40.1 ± 5.8 years, 56.8% female). Participants wore a Fitbit activity monitor, weighed themselves daily, and completed eight online surveys on socio-demographic characteristics. Participants also recalled their use of weight management strategies over the past month, at 8 timepoints during the 12-month study period.

**Results:**

Most participants (81%) reported using at least one weight management strategy, with exercise/physical activity being the most common strategy at each timepoint (40–54%). Those who accepted their current bodyweight were less likely to use at least one weight management strategy (Odds ratio = 0.38, 95% CI = 0.22–0.64, p < 0.01) and those who reported being physically active for weight maintenance had a greater reduction in bodyweight, than those who did not (between group difference: -1.2 kg, p < 0.01). The use of supplements and fasting were associated with poorer mental health and quality of life outcomes (p < 0.01).

**Conclusions:**

The use of weight management strategies appears to be common. Being physically active was associated with greater weight loss. Individuals who accepted their current body weight were less likely to use weight management strategies. Fasting and the use of supplements were associated with poorer mental health. Promoting physical activity as a weight management strategy appears important, particularly considering its multiple health benefits.

**Supplementary Information:**

The online version contains supplementary material available at 10.1186/s12889-023-16277-4.

## Background

Overweight and obesity is a growing, global public health issue, with high rates in high-, middle- and low-income countries [1–3]. Worldwide, 52% of adults aged 18 years and over are overweight or obese, with worldwide prevalence almost tripling between 1975 and 2016 [3]. Over these years in Australia, the prevalence of overweight and obesity in adults increased from 57 to 67%, accounting for 8.4% of the total burden of disease, which is a measure of the years of healthy life lost from living with ill health or dying prematurely from disease and injury [4]. These rates are of public health concern because individuals who are overweight or obese are at increased risk of various comorbid conditions, including cardiovascular disease, gastrointestinal disorders, type 2 diabetes, joint and muscular disorders, respiratory problems, psychological issues, morbidity, mortality, and higher health care costs [5–9].

The increased prevalence of overweight and obesity has contributed to an increased need for effective weight management strategies [10]. A previous systematic review of observational and intervention studies found that, while the strength of evidence is low for all weight management strategies, beneficial strategies include dietary changes (e.g., low-fat diets, eating less fast-food, eating more fruits and vegetables), as well as monitoring intensity during exercise, and participation in group lifestyle sessions with reminder text messages [11]. Cross-sectional findings from a sample of Australian general practitioner patients (n = 1335 adults) reported that 50% had tried to lose weight in the previous 12 months [12]. Of those, 72% reported changing their diet, 54% increased their physical activity or exercise, 7.5% used a professional weight loss program, 6.5% used over-the-counter weight loss supplements and 1.7% used medication [12]. Further, findings from a population-based prospective cohort (the Australian Longitudinal Study of Women’s Health) found that 74% of women (aged 45–52 years) actively used at least one strategy to manage their weight, with decreasing food quantity, cutting down on fats and sugars, using a commercial weight loss program, and exercise associated with preventing weight gain in 2007 [13]. However, these studies of various weight control strategies either did not involve assessment of associated changes in bodyweight [12] or evaluated women only [13]. Therefore, the aims of this study are to: (i) describe the weight management strategies used by a sample of Australian adults; (ii) examine the psychosocial and demographic characteristics of those who used each strategy; and (iii) examine whether use of weight management strategies was associated with weight change over a 12-month period.

## Methods

### Study design

The “Annual Rhythms In Adults’ lifestyle and health” (ARIA) [14] study was a prospective cohort study that followed participants over a 12-month period to assess daily activity, dietary intake, weight and wellbeing. The study protocol has been registered on the Australian New Zealand Clinical Trial Registry (ACTRN12619001430123, 16/10/2019). The study was approved by the University of South Australia Human Research Ethics committee (Protocol number: 201,901). Participants provided written informed consent prior to enrolment and this project was conducted in accordance with the Declaration of Helsinki [15].

### Participants and procedure

A community-based sample of 375 healthy adults, defined as not experiencing or receiving treatment for any life-threatening condition impacting daily lifestyle and health, was recruited from the greater metropolitan Adelaide area, South Australia. Participants were either parents of children who were enrolled in a previous cohort study evaluating children’s lifestyles (Life on Holidays Study [16]; cohorts 1 and 2, N = 119), or were parents of primary school children recruited from the community. An additional cohort (cohort 3, N = 256) of parents of primary school children were recruited from the community to reach the study’s target sample. Recruitment involved social media, community notice boards and mainstream media (cohort 3, N = 256). Recruitment involved 2 waves: Cohort 1 commenced data collection on December 1st, 2019, and cohorts 2 and 3 commenced data collection on December 1st, 2020. Eligibility criteria were: (i) 18 to 65 years old; (ii) living in greater metropolitan Adelaide, Australia; (iii) access to a Bluetooth-enabled mobile device or computer with home internet; (iv) proficiency in English; and (v) ambulant. Participants were excluded if they were (i) pregnant, (ii) had an implanted electronic medical device, or (iii) they were experiencing or receiving treatment for any life-threatening condition impacting daily lifestyle and health. An in-person home visit was conducted at baseline where the research staff gave participants a Fitbit Charge 3 activity monitor and Aria 2 or Aria Air body weight scale (Fitbit Inc, San Francisco, CA, USA). Participants were requested to wear the activity monitor and weigh themselves daily for the 12-month study period. In addition, they were asked to complete eight online surveys regarding their dietary intake, work status, recreational activities, weight loss intention, use of weight management strategies and wellbeing in the past month (timepoints: 1: mid-December; 2: mid-January; 3: mid-March; 4: mid-April; 5: mid-June; 6: mid-August; 7: mid-October; 8: mid-December [the following year]). Height measurements were also undertaken, and participants completed a self-report baseline demographics, health and lifestyle survey.

### Variables

#### Demographics

Demographic characteristics were reported at baseline. These included date of birth, sex, country of birth, marital status (never married, widowed, divorced, separated, married), number of children at home, highest education level (below year 10, year 10, year 11, year 12 or equivalent, certificate III/IV, advanced diploma/diploma, bachelor degree, postgraduate or higher degree), gross household income (AUD; *<*$50,000, $50,000-$99,999, $100,000-$199,999, >$200,000), occupation (open-ended response classified according to the Australian and New Zealand Standard Classification of Occupations [17]), hours worked per week (none, *<* 15, 15–35, 36+) and smoking status (yes, no). Height was measured at the baseline home visit (Leister Height Measure MKII).

#### Body weight

Body weight was assessed using Fitbit Aria 2 smart scales (Fitbit Inc, San Francisco, CA, USA) [18, 19]. Participants were instructed to weigh themselves daily in the morning, wearing minimal clothing, prior to meals and after voiding. Body weight data were collected remotely using our Fitnesslink software. This software was purpose-built for this study by software development company, Portal Australia, Adelaide, Australia. Fitnesslink was used to access participants’ Fitbit user account data, including user profile details, sleep, activity and weight data, and device data (battery status and time of most recent sync). The software automatically harvested the Fitbit weight data, removing the risk of data errors and reducing the risk of reactivity associated with using a participant logbook to collect weight data. Weight at baseline and 12 months was calculated as the mean of all measures taken over 14-day periods at baseline and at 12 months.

#### Weight change, acceptance, and management strategies

Weight management strategy items were adapted from items used in the Behavioral Risk Factor Surveillance System, collaboration with the Center for Disease Control and Prevention [20]. At baseline, participants were asked whether their weight was stable in the previous three months (“Yes, my weight has been stable [remained within 5% on bodyweight]”, “No, my weight has increased [by ≥ 5%]”, “No, my weight has decreased [by ≥ 5%]”) and whether their weight was acceptable to them. That is, whether an individual accepts their weight, regardless of whether they are an acceptable weight, underweight, or overweight [21, 22].

Data on use of weight management strategies were obtained using a self-report survey at each of the eight time points during the 12 months (Timepoints: 1: mid-December; 2: mid-January; 3: mid-March; 4: mid-April; 5: mid-June; 6: mid-August; 7: mid-October; 8: mid-December [the following year]. Participants responded ‘yes’ or ‘no’ to the following questions: In the past 30 days have you used any of the following to manage your weight: i) restricted calories (i.e., specifically made an effort to reduce calorie intake); ii) counted calories (i.e., monitored calorie intake, but did not specifically reduce or increase calorie intake); iii) exercised or been physically active; iv) taken diet pills; v) used special products such as powdered supplements; vi) fasted; vii) caused self to vomit after eating; or viii) anything else to lose or maintain weight. Participants were advised on the differences between restricting (i.e., specifically made an effort to reduce calorie intake) and counting calories (i.e., monitored calorie intake, but did not specifically reduce or increase calorie intake) when completing the survey.

#### Wellbeing

Quality of life was measured using the WHO Quality of Life assessment 26-item version (WHOQOL-BREF) at each of the eight timepoints [23]. WHOQOL-BREF is a self-report questionnaire with domains: physical health, psychological health, social relationships and environment. Of note, the environment component of the WHOQOL-BREF assesses an individual’s perception of their physical environment, including their satisfaction with their living conditions, access to healthcare services, transportation, safety, and other environmental factors that can influence their quality of life [23]. The WHOQOL-BREF has good discriminant validity, content validity and test-retest reliability and internal consistency [23, 24]. Symptoms of depression, anxiety and stress were assessed using the 21-item short-form Depression Anxiety Stress Scale (DASS-21) [25]. The DASS-21 has good convergent and discriminant validity, adequate construct validity, and high reliability [25–27].

### Statistical analysis

Baseline demographic characteristics and use of weight management strategies were reported using means and standard deviations for continuous data or counts and percentages for categorical data. The relationship between weight management strategies and change in weight over the 12-month period was assessed using linear mixed-effect models with random intercept for household (i.e., whether participants were from the same household, to account for the structure of the data, as some participants were from the same household), and fixed effects for age, sex, income, and baseline weight, and reported as coefficients and 95% confidence intervals (CIs). Univariate multinomial logistic regression was used to evaluate associations between the use of weight management strategies and psycho-sociodemographic characteristics. Missing data analysis was performed to assess the extent and nature of missingness in the dataset. Since the dataset was largely complete and the amount of missing data was low, imputation methods were not utilized, and the analyses were conducted using the available data. A graph of the proportion of total participants using each weight management strategy (expressed as a proportion of the sample size at each timepoint) was created using Microsoft Excel. Variables for which the p-value was < 0.05 in univariate analyses were included in multivariable models using multinomial regression and reported as odds ratios (OR) and 95% CI. Holm-Bonferroni adjustments for multiple testing were performed. This analysis involved conducting a secondary analysis of existing data, therefore, formal sample size calculations were not performed. All analyses were conducted using SPSS, version 25 (IBM, NY, USA).

## Results

### Participant characteristics

A total of 375 participants were recruited into the study, of whom 7 formally withdrew during the 12-month study period (1.8% drop-out). Their baseline characteristics are shown in Table 1. Just over half the participants were female and half were aged 39 years or less. Participants were predominantly born in Australia and married or living in a relationship. Of the 368 participants who remained enrolled in the study at 12 months, survey data on the use of weight management strategies was available for n = 350 (95.1%), n = 343 (93.2%), n = 344 (93.5%), n = 334 (90.8%), n = 317 (86.1%), n = 327 (88.9%), n = 319 (86.7%), n = 320 (87.0%) at time points 1 to 8, respectively. At baseline, mean body weight was 83.8 kg, and most participants were classed as overweight or obese based on body mass index. Most participants (77%) reported that their weight was stable prior to study enrolment. Weight data at 12 months was available for 368 participants.

**Table 1:** Baseline characteristics of participants

	Used any strategy (at least once) at any point					
	Yes, n = 297		No, n = 71		Total, n = 368	
Variable	Mean or n	SD or %	Mean or n	SD or %	Mean or n	SD or %
Age, years	40.2	5.8	39.8	5.9	40.1	5.8
<39 years	148	49.8%	37	52.1%	185	50.3%
≥40 years	149	50.2%	34	47.9%	183	49.7%
Sex						
Female	174	58.6%	35	50.7%	209	56.8%
Male	123	41.4%	36	49.3%	159	43.2%
Weight, kg	84.6	20.5	80.8	20.0	83.8	20.4
Body mass index, kg/m^2^	29.4	7.9	28.0	7.7	29.1	7.9
Underweight	11	3.7%	5	7.0%	16	4.3%
Normal	82	27.6%	23	32.4%	105	28.5%
Overweight	90	30.3%	20	28.2%	110	29.9%
Obese	114	38.4%	23	32.4%	137	37.2%
Country of birth						
Australia	223	75.1%	57	80.3%	280	76.1%
Other	74	24.9%	14	19.7%	88	23.9%
Marital status						
Married	43	14.5%	12	16.9%	313	85.1%
Other	254	85.5%	59	83.1%	55	14.9%
Education						
Some or completed high school	66	17.9%	53	17.8%	13	18.3%
Trade school or certificate	125	34.0%	100	33.7%	25	35.2%
University (bachelor or higher degree)	177	48.1%	144	48.5%	33	46.5%
Income						
<$50,000	27	9.1%	10	14.1%	37	10.1%
$50,000 to$99,999	90	30.3%	21	29.6%	111	30.2%
$100,000 to $199,999	141	47.5%	35	49.3%	176	47.8%
>$200,000	39	13.1%	5	7.0%	44	12.0%
Smoker, yes	24	8.1%	11	15.5%	35	9.5%
Weight stable in last 3 months? Yes	222	74.7%	60	84.5%	282	76.6%
No, increased	56	18.9%	7	9.9%	63	17.1%
No, decreased	19	6.4%	4	5.6%	23	6.3%
Weight acceptance						
Acceptable weight	102	34.3%	41	57.7%	143	38.9%
Underweight	0	0.0%	2	2.8%	2	0.5%
Overweight	195	65.7%	28	(39.4%	223	60.6%

Baseline characteristics of total sample (n = 368), and stratified based on use of weight management strategies (yes, n = 297; no = 71) during the 12-month period

### Use of weight control strategies

**Fig. 1:**
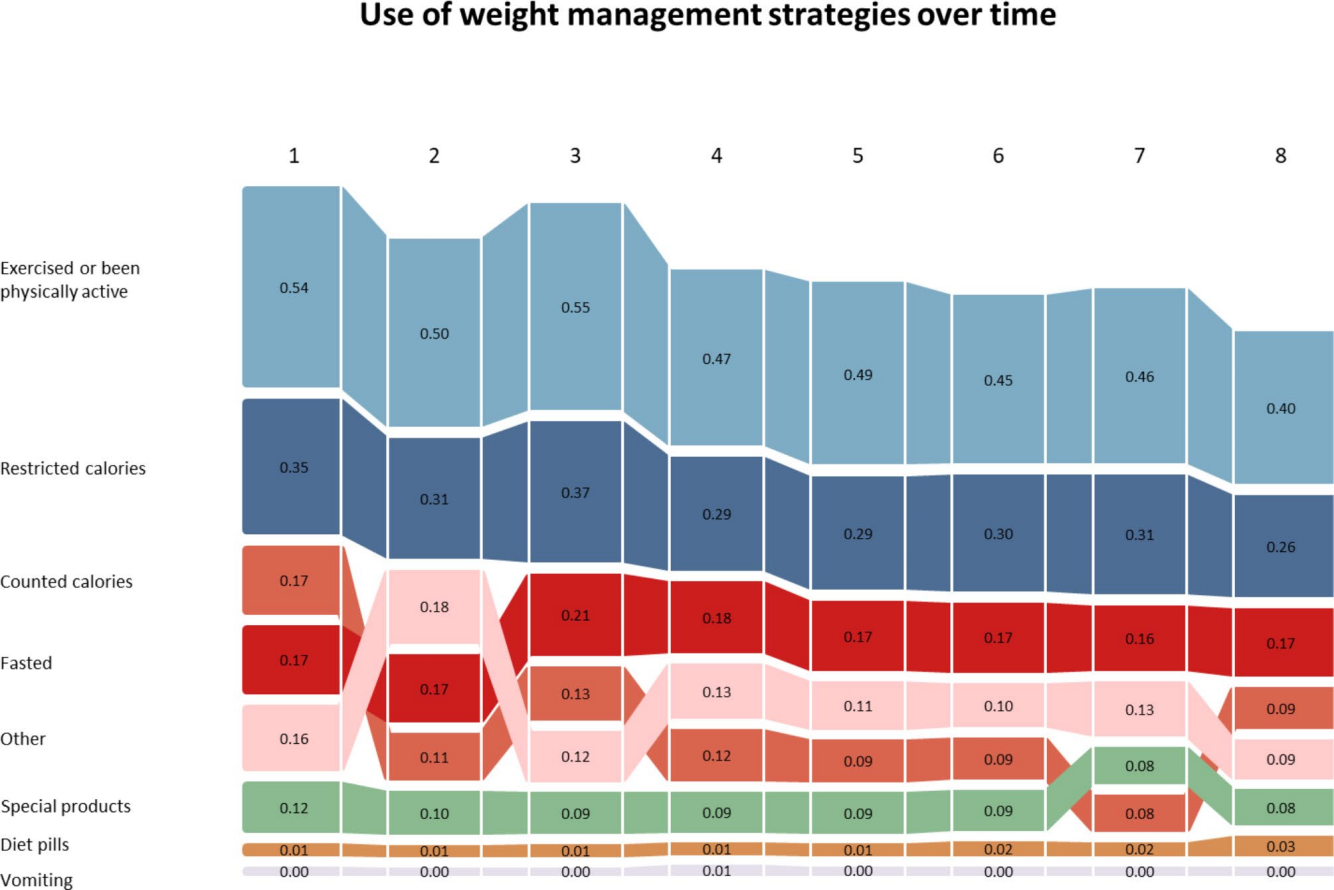
Graph of changes in the use of each weight management strategy over the 8 timepoints, expressed as a proportion of the sample size at each time point (Timepoints: 1: mid-December; 2: mid-January; 3: mid-March; 4: mid-April; 5: mid-June; 6: mid-August; 7: mid-October; 8: mid-December [the following year]) Change in the use of each weight management strategy over eight timepoints (as a proportion of the sample size at each timepoint)

An overview of the use of weight control strategies is shown in Table 2. The majority of participants (81%) reported using at least one weight management strategy. Change in the use of each weight management strategy over eight timepoints (as a proportion of the sample size at each timepoint) is shown in Fig. 1. Exercising or being physically active was the most commonly reported strategy at each timepoint, ranging from 54% at timepoint 1, to 40% at timepoint 8.

**Table 2:** Use of weight control strategies over the 12-month period (n = 368)

Variable	Yes^1^ n (%)	No^2^ n (%)
Used any strategy (at least once) at any point	297 (80.7%)	71 (19.3%)
Restricted calories	230 (62.5%)	138 (37.5%)
Counted calories	103 (28.0%)	265 (72.0%)
Exercised or been physically active	282 (76.6%)	86 (23.4%)
Taken diet pills	21 (5.7%)	347 (94.3%)
Used special products such as powdered supplements	88 (23.9%)	280 (76.1%)
Fasted	150 (40.8%)	218 (59.2%)
Caused self to vomit after eating	5 (1.4%)	363 (98.6%)
Anything else to lose or maintain weight	131 (35.6%)	237 (64.4%)

^1^ Used the weight management strategy at least once over the 8 timepoints

^2^Did not use the weight management strategy over the 8 timepoints

### Relationship between weight management strategies and change in weight over 1 year

The relationships between the use of each weight management strategy and changes in weight are shown in Table 3. Almost none of the weight management strategies were significantly associated with weight change, with the exception of exercising or being physically active. Being physically active or exercising was associated with a greater reduction in body weight than not being physically active (between group difference: -1.2 kg, 95% CI: -2.3, -0.1 kg; p < 0.01).

**Table 3:** Effect of weight change strategies on change in weight over 12 months

Weight change (kg) after 12 months			
	Estimate	95% CI	p-value
Use any strategy (at least once) at any point			
Yes	-1.1	-2.3, 0.1	0.063
No	REF		
Restricted calories^1^			
Yes	-0.3	-1.3, 0.7	0.517
No	REF		
Counted calories^1^			
Yes	0.1	-1.0, 1.1	0.916
No	REF		
Exercised or been physically active^1^			
Yes	-1.2	-2.3, -0.1	0.034
No	REF		
Taken diet pills^1^			
Yes	0.2	-1.9, 2.3	0.839
No	REF		
Used special products such as powdered supplements^1^			
Yes	0.4	-0.8, 1.5	0.532
No	REF		
Fasted^1^			
Yes	-0.4	-1.4, 0.5	0.371
No	REF		
Caused self to vomit after eating^1^			
Yes	-2.8	-6.9, 1.3	0.176
No	REF		
Anything else to lose or maintain weight^1^			
Yes	-0.4	-1.4, 0.6	0.460
No	REF		

The relationships between the use of each weight management strategy and changes in weight

^1^ Estimates from linear mixed effect models with random intercept for household, and fixed effects for age, sex, income, and baseline weight,

^2^ Used the strategy at least once over the 12 months

### Association between use of weight management strategies and psycho-sociodemographic characteristics

Association between the use of each weight change strategy and psycho-sociodemographic characteristics are shown in Tables S1-S15. Compared with not accepting one’s bodyweight, the odds of using any weight management strategy over the 12 months was lower in those that accepted their bodyweight (OR = 0.38, 95% CI = 0.22, 0.64, p < 0.01). Compared with having a stable weight in the three months prior to study enrolment, the odds of restricting calories (OR = 0.39, 95% CI = 0.20, 0.74, p < 0.01) and fasting (OR = 0.44, 95% CI = 0.25, 0.77, p < 0.01) were lower in those who increased weight in the three months prior. Compared with not accepting one’s bodyweight, the odds of restricting calories (OR = 0.52, 95% CI = 0.33, 0.80, p < 0.01) and fasting (OR = 0.49, 95% CI = 0.32, 0.77, p < 0.01) were lower in those that accepted their bodyweight. Compared with females, the odds of counting calories (OR = 0.49, 95% CI = 0.30, 0.79, p < 0.01) and using other weight management strategy (OR = 0.41, 95% CI = 0.26, 0.65, p < 0.01) were lower in males. Use of special products (e.g., powders) was positively associated with baseline weight (b = 0.02, SE = 0.00, p < 0.01) and depression (b = 0.04, SE = 0.01, p < 0.01), and negatively associated with physical QOL (b=-0.15, SE = 0.01, p < 0.01) and psychological QOL (b=-0.14, SE = 0.04, p < 0.01). Fasting was negatively associated with psychological QOL (b=-0.17, SE = 0.04, p < 0.01) and environmental QOL (b=-0.19, SE = 0.05, p < 0.01). Further, compared with having a comorbid condition, the odds of fasting were lower than in those without a comorbid condition (OR = 0.34, 95% CI = 0.21, 0.53, p < 0.01).

## Discussion

The aims of this study were to describe the weight management strategies used by a sample of Australian adults and assess whether the use of weight management strategies over a 12-month period was associated with weight loss and psycho-sociodemographic characteristics. The findings showed that weight management strategies were commonly used, with 81% of participants using at least one strategy over the 12 months. Exercise/physical activity was the most common strategy at each timepoint and was associated with a -1.2 kg greater reduction in body weight than not being physically active. Other key findings were: (1) those that accepted their weight had a lower odds of using any weight management strategy, (2) the odds of restricting calories and fasting were lower in those who increased weight prior to enrolment, than those who maintained weight, (3) the odds of restricting calories and fasting was lower in those that accepted their bodyweight, than those who didn’t accept their bodyweight, and (4) the odds of counting calories and using any other weight management strategy was lower in males than females.

The use of weight management strategies was common, with 77% being physically active or exercising, 63% restricting calories, 41% fasting, 28% counting calories, 24% using supplements (e.g., powders), 6% using diet pills and 1% vomiting. These findings are reasonably comparable to a previous population-based cohort study of Australian women [28], where the use of various weight management strategies among those who had been on a diet in the past year included cutting down fats/sugars (82%), reducing meal size (81%), exercise (60%), using a commercial weight loss program (19%), fasting (13%), meal replacements or slimming products (6%), laxatives, diuretics and diet pills (3%), vomiting (1.9%). Our sample appeared to use exercise and fasting at slightly higher rates than the previous study (exercise 80% vs. 60% and fasting 41% vs. 13%). This observation might be due to ongoing public health effects promoting the benefits of physical activity for health [29], and a growing interest in fasting practices, such as intermittent fasting [30], in more recent times.

Our findings suggest that the use of weight management strategies decreased over the 12 months (from mid-December to mid-December the following year; exercise or being physically active: 54 to 40%; restricted calories 35 to 26%; counted calories: 17 to 9%; other: 16 to 9%; use of special products: 12 to 8%). All of the weight management strategies appeared to fall at a similar rate, with perhaps the exception of calorie-counting, which appeared to decline at a greater rate. There is evidence to suggest that people who are able to lose weight and keep it off for at least 3 months are more likely to be successful in the long term [31]. However, many individuals do not sustain weight control behaviour long term (i.e., for 12 months or more) [32]. A previous prospective cohort study which evaluated use of weight management strategies over 4 years among 1120 US adults found that the median duration of use for most strategies was 10 months for decreasing fat intake, and 7 months for increased physical activity, over the 4 years [32]. An alternative explanation may be measurement bias - participants in our study were asked to complete the survey at eight times points across the 12-month period. It is possible that measurement fatigue is behind the gradual reduction in reporting over weight management strategies across the study period.

At each timepoint, exercising or being physically active was the most reported weight management strategy, ranging from 54% at timepoint 1, to 40% at timepoint 8. Further, the findings indicated that those who were physically active or exercised, reduced their bodyweight by -1.21 kg more, than those who were not physically active. Whilst weight maintenance is a recognised benefit of physical activity, most research comparing the relative benefits of diet versus exercise for weight loss identify diet as the more potent strategy [33, 34]. Therefore, it was somewhat surprising that physical activity was associated with weight loss in this study, while dietary strategies were not. Findings from previous systematic reviews of randomised controlled trials have found that physical activity combined with diet and behavioural components leads to greater weight loss at 12 months, compared with diet-only or physical activity-only interventions, weight loss mean difference range − 1.17 to 3.02 kg [35, 36]. The popularity of physical activity for weight management reported in this study, and that the use of physical activity appeared to be a more effective weight management strategy, may suggest that physical activity may be a particularly achievable and acceptable weight management strategy (particularly given that physical activity is associated with immediate psychological and cognitive benefits, such as improved mood and vitality [37]).

Weight acceptance was associated with use of weight management strategies, with those that didn’t accept their weight being more likely to use weight management strategies, than those who accepted their bodyweight. These findings are consistent with previous work that has shown that adults who perceive themselves as overweight being more likely to attempt to lose weight [38], and more likely to report using exercise as a weight control strategy than those who do not perceive themselves as overweight [39]. In addition, findings from a recent systematic review showed strong evidence for an association between perceived overweight and weight loss attempts; individuals who perceived themselves as overweight had a higher likelihood of intending or attempting to lose weight than those who perceived themselves as normal weight [40]. Furthermore, individuals who identify as overweight experience higher levels of body dissatisfaction [41] and may therefore have a greater desire to lose weight than individuals who do not identify as overweight. However, longitudinal studies of adolescents and adults have shown that perceiving oneself as overweight is associated with greater long-term weight gain in individuals with both measured normal weight and measured overweight (i.e., measured body mass index using objective methods) [42, 43] therefore, perception of overweight may not necessarily be associated with effective long-term weight management.

Consistent with previous findings [44], our present findings showed that males were less likely to restricted calories, compared with females. In a previous study by Harring et al. [44], higher proportions of US college-aged women reported trying to lose weight compared with men (61% vs. 34%, respectively). Harring et al. [44] also reported higher proportions of women were using the following weight management strategies: exercise (63% vs. 44%), diet (42% vs. 22%), vomiting after meals (4% vs. 0.7%), diet pills (5% vs. 2%), diet and exercise (36% vs. 19%), compared with men, respectively. With the exception of restricting calories, our findings showed no sex differences for the use of counting calories, exercising or being physically active, diet pills, use of special products (such as powdered supplements), fasting and self-vomiting. Previous findings suggest that being a parent can influence attitudes and practices in weight management strategies. For example, a study found that parents of minor children had poorer weight loss outcomes and behavioural adherence, than participants without children, in a rural community-based weight loss intervention [45]. Therefore, the differences in our study compared with Harring et al. [44] is likely attributed to the difference samples (community-dwelling adults who were parents of school-aged children, versus college students). In addition, there was some evidence to suggest that fasting, and the use of special powders or supplements (to manage weight), were associated with worse depression and QOL. It is possible that people who try fasting and using supplements to lose weight may have failed previously to lose weight and are looking for alternative methods to achieve weight loss [46]. Prior work has indicated that repeated failed attempts to manage weight are associated with reductions in psychological well-being [47]. While others have reported that worse psychological well-being is associated with weight gain [48]. Therefore, future research is required to understand the interrelationships between use of weight management strategies, weight changes and wellbeing.

### Strengths and limitations

Strengths of this study were that the sample was reasonably reflective of middle-aged Australian adults (in terms of sex, household structure, income, weight status), weight was objectively measured, and retention and data completeness were high. Limitations of this work was that participants were from one Australian city, were all parents, the sample size was modest, and the use of a non-validated questionnaire to assess weight acceptance. Sample size calculations were conducted for the primary analysis [14]. Our analysis involved conducting a secondary analysis of existing data, and as such, formal sample size calculations were not performed. Therefore, the study may have limited power to detect relationships (particularly if they are small in magnitude) and the generalisability of findings to other geographical regions and demographic groups are unclear. An additional limitation is the observational study design which limits the ability to infer causality.

### Implications

Given the high, and increasing, rates of overweight and obesity in Australia and many other countries around the world, effective weight management strategies are needed. Our present findings identified physical activity and exercise as the most popular weight management strategy, and the single weight management strategy associated with weight loss at 12 months. This finding supports current national and international weight management guidelines which recommend exercise and healthy eating patterns [49]. It is encouraging, given that physical activity confers many other health benefits in addition to its benefits for weight control [50].

## Conclusion

Overall, the use of weight management strategies was common in this sample of Australian adults. The most popular weight management strategies included exercising or being physically active, restricting calories, and fasting. Those who didn’t accept their current bodyweight were more likely to use at least one weight management strategy and those who were reported being physically active for weight maintenance had a greater reduction in bodyweight, than those who did not. Public health weight management approaches should include weight management strategies that are associated with effective weight management, with our findings indicating that physical activity and exercise is a popular weight management strategy and is also associated with weight loss at 12 months.

## Electronic supplementary material

Below is the link to the electronic supplementary material.

Supplementary Material 1

## Data Availability

The datasets used and/or analysed during the current study are available from the corresponding author on reasonable request.
