# Supplementary material for: Association between the use of weight management strategies and weight change among Australian adults over 12 months: an observational study
Source: BMC Public Health. 2023 Jul 31;23:1461. doi: 10.1186/s12889-023-16277-4 (PMC10391811; doi:10.1186/s12889-023-16277-4)
Supplement: Supplementary file 1 — Supplementary Material 1 [file 12889_2023_16277_MOESM1_ESM.docx]

Supplementary tables

Table S1. Association between use of weight change strategies over the 12 months and psycho-sociodemographic characteristics, results of univariate analyses. 2

Table S2. Association between use of weight change strategies over the 12 months and psycho-sociodemographic characteristics, results of multivariate analyses. 4

Table S3. Association between restricting calories and psycho-sociodemographic characteristics, results of univariate analyses. 5

Table S4. Association between restricting calories and psycho-sociodemographic characteristics, results of multivariate analyses. 7

Table S5. Association between counting calories and psycho-sociodemographic characteristics, results of univariate analyses. 8

Table S6. Association between counting calories and psycho-sociodemographic characteristics, results of multivariate analyses. 10

Table S7. Association between exercise and physical activity and psycho-sociodemographic characteristics, results of univariate analyses. 11

Table S8. Association between taking diet pills and psycho-sociodemographic characteristics, results of univariate analyses. 13

Table S9. Association between use special products and psycho-sociodemographic characteristics, results of univariate analyses. 15

Table S10. Association between use special products and psycho-sociodemographic characteristics, results of multivariate analyses. 17

Table S11. Association between fasting and psycho-sociodemographic characteristics, results of univariate analyses. 18

Table S12. Association between fasting and psycho-sociodemographic characteristics, results of multivariate analyses. 20

Table S13. Association between self-vomiting and psycho-sociodemographic characteristics, results of univariate analyses. 21

Table S14. Association between use of any other weight change strategies and psycho-sociodemographic characteristics, results of univariate analyses. 23

Table S15. Association between use of any other weight change strategies and psycho-sociodemographic characteristics, results of multivariate analyses. 25

Table S1. Association between use of weight change strategies over the 12 months and psycho-sociodemographic characteristics, results of univariate analyses.

| Used any weight control strategy at least once over the 12 months  Yes (reference category is No) | | | | |
| --- | --- | --- | --- | --- |
|  | OR | 95% CI | p-value | Adjusted p-value^1^ |
| Age (years)  <39 years  ≥40 years | 0.91  REF | 0.54, 1.53 | 0.73 | 1.00 |
| Gender  Male  Female | 0.68  REF | 0.40, 1.15 | 0.15 | 1.00 |
| Country of birth  Australia  Other | 0.74  REF | 0.39, 1.40 | 0.35 | 1.00 |
| Marital status  Married or defacto  Other | 1.20  REF | 0.59, 2.41 | 0.60 | 1.00 |
| Income  ≤$99,999  >$99,999 | 0.83  REF | 0.49, 1.41 | 0.51 | 1.00 |
| Smoker (yes)  No  Yes | 2.08  REF | 0.96, 4.48 | 0.06 | 1.00 |
| Weight stable in last 3 months?  No, increased  No, decreased  Yes | 0.46  0.66  REF | 0.20, 1.06  0.25, 2.37 | 0.07 | 1.00 |
|  |  |  | 0.66 | 1.00 |
| Weight acceptance  Yes  No | 0.38  REF | 0.22, 0.64 | <0.01 | <0.01 |
| Presence of comorbid conditions  No  Yes | 0.68  REF | 0.40, 1.15 | 0.15 | 1.00 |
| Weight  Baseline  Change over 12m | 0.01  -0.05 | <0.01  0.03 | 0.16 | 1.00 |
|  |  |  | 0.04 | 0.96 |
| QOL - Physical domain  Baseline  Change over 12m | -0.15  0.10 | 0.06  0.08 | 0.01 | 0.25 |
|  |  |  | 0.20 | 1.00 |
| QOL - Psychological domain  Baseline  Change over 12m | -0.10  0.10 | 0.05  0.08 | 0.07 | 1.00 |
|  |  |  | 0.18 | 1.00 |
| QOL - Social relationships domain  Baseline  Change over 12m | -0.05  0.06 | 0.04  0.05 | 0.22 | 1.00 |
|  |  |  | 0.27 | 1.00 |
| QOL - Environment domain  Baseline  Change over 12m | -0.06  0.16 | 0.07  0.08 | 0.33 | 1.00 |
|  |  |  | 0.05 | 1.00 |
| Depression  Baseline  Change over 12m | -0.01  -0.01 | 0.01  0.02 | 0.92 | 0.92 |
|  |  |  | 0.82 | 1.00 |
| Anxiety  Baseline  Change over 12m | 0.01  0.01 | 0.02  0.03 | 0.71 | 1.00 |
|  |  |  | 0.70 | 1.00 |
| Stress  Baseline  Change over 12m | 0.01  -0.04 | 0.01  0.02 | 0.63 | 1.00 |
|  |  |  | 0.10 | 1.00 |
| ^1^ P-values adjusted using Holm-Bonferroni correction method | | | | |

Table S2. Association between use of weight change strategies over the 12 months and psycho-sociodemographic characteristics, results of multivariate analyses.

| Used any weight control strategy at least once over the 12 months  Yes (reference category is No) | | | |
| --- | --- | --- | --- |
|  | B | SE | p-value |
| Intercept | 1.87 | 0.19 | <0.01 |
|  | OR | 95% CI | p-value |
| Weight acceptance  Yes  No | 0.38  REF | 0.22, 0.64 | <0.01 |

Table S3. Association between restricting calories and psycho-sociodemographic characteristics, results of univariate analyses.

| Restricted calories  Yes (reference category is No) | | | | |
| --- | --- | --- | --- | --- |
|  | OR | 95% CI | p-value | Adjusted p-value^1^ |
| Age (years)  <39 years  ≥40 years | 0.77  REF | 0.50, 1.17 | 0.22 | 1.00 |
| Gender  Male  Female | 0.81  REF | 0.53, 1.24 | 0.34 | 1.00 |
| Country of birth  Australia  Other | 1.28  REF | 0.78, 2.09 | 0.31 | 1.00 |
| Marital status  Married or defacto  Other | 0.94  REF | 0.52, 1.71 | 0.85 | 1.00 |
| Income  ≤$99,999  >$99,999 | 0.97  REF | 0.63, 1.50 | 0.91 | 0.91 |
| Smoker (yes)  No  Yes | 1.65  REF | 0.82, 3.33 | 0.15 | 1.00 |
| Weight stable in last 3 months?  No, increased  No, decreased  Yes | 0.39  0.38  REF | 0.20, 0.74  0.13, 1.05 | <0.01 | <0.01 |
|  |  |  | 0.06 | 1.00 |
| Weight acceptance  Yes  No | 0.52  REF | 0.33, 0.80 | <0.01 | <0.01 |
| Presence of comorbid conditions  No  Yes | 0.77  REF | 0.50, 1.18 | 0.23 | 1.00 |
| Weight  Baseline  Change over 12m | 0.01  -0.02 | 0.01  -0.02 | 0.05 | 1.00 |
|  |  |  | 0.37 | 1.00 |
| QOL - Physical domain  Baseline  Change over 12m | -0.12  0.04 | 0.04  0.06 | 0.01 | 0.23 |
|  |  |  | 0.52 | 1.00 |
| QOL - Psychological domain  Baseline  Change over 12m | -0.14  0.08 | 0.04  0.06 | 0.02 | 0.44 |
|  |  |  | 0.20 | 1.00 |
| QOL - Social relationships domain  Baseline  Change over 12m | -0.09  0.04 | 0.03  0.04 | 0.01 | 0.23 |
|  |  |  | 0.33 | 1.00 |
| QOL - Environment domain  Baseline  Change over 12m | -0.04  0.09 | 0.05  0.07 | 0.41 | 1.00 |
|  |  |  | 0.16 | 1.00 |
| Depression  Baseline  Change over 12m | 0.02  0.01 | 0.01  0.01 | 0.11 | 1.00 |
|  |  |  | 0.83 | 1.00 |
| Anxiety  Baseline  Change over 12m | 0.03  0.01 | 0.02  0.02 | 0.07 | 1.00 |
|  |  |  | 0.78 | 1.00 |
| Stress  Baseline  Change over 12m | 0.01  -0.01 | 0.01  0.01 | 0.30 | 1.00 |
|  |  |  | 0.29 | 1.00 |
| ^1^ P-values adjusted using Holm-Bonferroni correction method | | | | |

Table S4. Association between restricting calories and psycho-sociodemographic characteristics, results of multivariate analyses.

| Restricted calories  Yes (reference category is No) | | | |
| --- | --- | --- | --- |
|  | B | SE | p-value |
| Intercept | 1.46 | 0.51 | <0.01 |
|  | OR | 95% CI | p-value |
| Weight stable  No, increased  No, decreased  Yes | 0.40  0.91  REF | 0.14, 1.12  0.28, 2.93 | 0.08  0.88 |
| Weight acceptance  Yes  No | 0.58  REF | 0.37, 0.90 | 0.01 |

Table S5. Association between counting calories and psycho-sociodemographic characteristics, results of univariate analyses.

| Counted calories  Yes (reference category is No) | | | | |
| --- | --- | --- | --- | --- |
|  | OR | 95% CI | p-value | Adjusted p-value^1^ |
| Age (years)  <39 years  ≥40 years | 0.95  REF | 0.60, 1.51 | 0.85 | 1.00 |
| Gender  Male  Female | 0.49  REF | 0.30, 0.79 | <0.01 | <0.01 |
| Country of birth  Australia  Other | 0.97  REF | 0.57, 1.65 | 0.92 | 1.00 |
| Marital status  Married or defacto  Other | 0.89  REF | 0.54, 1.98 | 0.89 | 1.00 |
| Income  ≤$99,999  >$99,999 | 1.09  REF | 0.68, 1.73 | 0.70 | 1.00 |
| Smoker (yes)  No  Yes | 0.96  REF | 0.44, 2.09 | 0.93 | 1.00 |
| Weight stable in last 3 months?  No, increased  No, decreased  Yes | 0.60  0.79  REF | 0.34, 1.08  0.31, 2.01 | 0.09 | 1.00 |
|  |  |  | 0.63 | 1.00 |
| Weight acceptance  Yes  No | 0.62  REF | 0.38, 1.01 | 0.05 | 1.00 |
| Presence of comorbid conditions  No  Yes | 0.90  REF | 0.56, 1.42 | 0.65 | 1.00 |
| Weight  Baseline  Change over 12m | 0.00  0.00 | 0.01  0.02 | 0.58 | 1.00 |
|  |  |  | 0.99 | 0.99 |
| QOL - Physical health domain score  Baseline  Change over 12m | -0.07  -0.02 | 0.04  0.06 | 0.14 | 1.00 |
|  |  |  | 0.71 | 1.00 |
| QOL - Psychological domain score  Baseline  Change over 12m | -0.10  0.00 | 0.04  0.06 | 0.02 | 0.50 |
|  |  |  | 0.93 | 1.00 |
| QOL - Social relationships domain score  Baseline  Change over 12m | -0.02  0.00 | 0.03  0.04 | 0.51 | 1.00 |
|  |  |  | 0.93 | 1.00 |
| QOL - Environment domain score  Baseline  Change over 12m | -0.04  0.04 | 0.05  0.07 | 0.49 | 1.00 |
|  |  |  | 0.54 | 1.00 |
| Depression  Baseline  Change over 12m | 0.02  0.01 | 0.01  0.02 | 0.15 | 1.00 |
|  |  |  | 0.45 | 1.00 |
| Anxiety  Baseline  Change over 12m | 0.03  -0.01 | 0.01  0.02 | 0.04 | 0.96 |
|  |  |  | 0.49 | 1.00 |
| Stress  Baseline  Change over 12m | 0.02  -0.01 | 0.01  0.01 | 0.06 | 1.00 |
|  |  |  | 0.46 | 1.00 |
| ^1^ P-values adjusted using Holm-Bonferroni correction method | | | | |

Table S6. Association between counting calories and psycho-sociodemographic characteristics, results of multivariate analyses.

| Counted calories 2  Yes (reference category is No) | | | |
| --- | --- | --- | --- |
|  | B | SE | p-value |
| Intercept | -0.66 | 0.14 | <0.01 |
|  | OR | 95% CI | p-value |
| Gender  Male  Female | 0.49  REF | 0.30, 0.79 | <0.01 |

Table S7. Association between exercise and physical activity and psycho-sociodemographic characteristics, results of univariate analyses.

| Exercised or been physically active  Yes (reference category is No) | | | | |
| --- | --- | --- | --- | --- |
|  | OR | 95% CI | p-value | Adjusted p-value^1^ |
| Age (years)  <39 years  ≥40 years | 0.95  REF | 0.58, 1.54 | 0.85 | 1.00 |
| Gender  Male  Female | 0.54  REF | 0.33, 0.89 | 0.01 | 0.38 |
| Country of birth  Australia  Other | 0.73  REF | 0.40, 1.32 | 0.30 | 1.00 |
| Marital status  Married or defacto  Other | 1.143  REF | 0.59, 2.21 | 0.69 | 1.00 |
| Income  ≤$99,999  >$99,999 | 0.91  REF | 0.56, 1.49 | 0.91 | 0.91 |
| Smoker (yes)  No  Yes | 1.57  REF | 0.73, 3.36 | 0.23 | 1.00 |
| Weight stable in last 3 months?  No, increased  No, decreased  Yes | 0.47  0.60  REF | 0.22, 1.01  0.19, 1.83 | 0.05 | 1.00 |
|  |  |  | 0.37 | 1.00 |
| Weight acceptance  Yes  No | 0.48  REF | 0.29, 0.79 | <0.01 | 0.10 |
| Presence of comorbid conditions  No  Yes | 0.62  REF | 0.38, 1.01 | 0.05 | 1.00 |
| Weight  Baseline  Change over 12m | 0.00  -0.06 | 0.00  0.028 | 0.47 | 1.00 |
|  |  |  | 0.03 | 0.72 |
| QOL - Physical health domain score  Baseline  Change over 12m | -0.09  0.09 | 0.05  0.07 | 0.09 | 1.00 |
|  |  |  | 0.20 | 1.00 |
| QOL - Psychological domain score  Baseline  Change over 12m | -0.02  0.10 | 0.05  0.07 | 0.61 | 1.00 |
|  |  |  | 0.17 | 1.00 |
| QOL - Social relationships domain score  Baseline  Change over 12m | -0.02  0.08 | 0.04  0.05 | 0.55 | 1.00 |
|  |  |  | 0.10 | 1.00 |
| QOL - Environment domain score  Baseline  Change over 12m | 0.02  0.16 | 0.06  0.08 | 0.72 | 1.00 |
|  |  |  | 0.05 | 1.00 |
| Depression  Baseline  Change over 12m | 0.00  -0.02 | 0.01  0.02 | 0.83 | 1.00 |
|  |  |  | 0.32 | 1.00 |
| Anxiety  Baseline  Change over 12m | 0.02  -0.00 | 0.02  0.03 | 0.38 | 1.00 |
|  |  |  | 0.75 | 1.00 |
| Stress  Baseline  Change over 12m | 0.01  -0.04 | 0.01  0.02 | 0.45 | 1.00 |
|  |  |  | 0.06 | 1.00 |
| ^1^ P-values adjusted using Holm-Bonferroni correction method | | | | |

Table S8. Association between taking diet pills and psycho-sociodemographic characteristics, results of univariate analyses.

| Taken diet pills  Yes (reference category is No) | | | | |
| --- | --- | --- | --- | --- |
|  | OR | 95% CI | p-value | Adjusted p-value^1^ |
| Age (years)  <39 years  ≥40 years | 3.37  REF | 1.20, 9.40 | 0.02 | 0.48 |
| Gender  Male  Female | 0.50  REF | 0.19, 1.33 | 0.17 | 1.00 |
| Country of birth  Australia  Other | 1.35  REF | 0.44, 4.14 | 0.59 | 1.00 |
| Marital status  Married or defacto  Other | 0.53  REF | 0.18, 1.53 | 0.24 | 1.00 |
| Income  ≤$99,999  >$99,999 | 0.91  REF | 0.36, 2.25 | 0.83 | 1.00 |
| Smoker (yes)  No  Yes | 0.99  REF | 0.22, 4.47 | 0.99 | 0.99 |
| Weight stable in last 3 months?  No, increased  No, decreased  Yes | 0.771  0.348  REF | 0.24, 2.42  0.09, 1.31 | 0.65 | 1.00 |
|  |  |  | 0.11 | 1.00 |
| Weight acceptance  Yes  No | 0.24  REF | 0.07, 0.85 | 0.02 | 0.62 |
| Presence of comorbid conditions  No  Yes | 0.92  REF | 0.38, 2.26 | 0.87 | 1.00 |
| Weight  Baseline  Change over 12m | 0.02  -0.00 | 0.00  0.04 | 0.03 | 0.66 |
|  |  |  | 0.90 | 1.00 |
| QOL - Physical health domain score  Baseline  Change over 12m | -0.15  -0.24 | 0.08  0.11 | 0.05 | 1.00 |
|  |  |  | 0.04 | 0.84 |
| QOL - Psychological domain score  Baseline  Change over 12m | -0.12  -0.17 | 0.08  0.12 | 0.12 | 1.00 |
|  |  |  | 0.16 | 1.00 |
| QOL - Social relationships domain score  Baseline  Change over 12m | 0.09  -0.11 | 0.07  0.08 | 0.22 | 1.00 |
|  |  |  | 0.18 | 1.00 |
| QOL - Environment domain score  Baseline  Change over 12m | -0.10  -0.09 | 0.11  0.13 | 0.33 | 1.00 |
|  |  |  | 0.48 | 1.00 |
| Depression  Baseline  Change over 12m | 0.05  -0.02 | 0.02  0.03 | 0.02 | 0.48 |
|  |  |  | 0.45 | 1.00 |
| Anxiety  Baseline  Change over 12m | 0.05  0.14 | 0.02  0.05 | 0.07 | 1.00 |
|  |  |  | <0.01 | 0.21 |
| Stress  Baseline  Change over 12m | 0.03  0.03 | 0.02  0.03 | 0.19 | 1.00 |
|  |  |  | 0.32 | 1.00 |
| ^1^ P-values adjusted using Holm-Bonferroni correction method | | | | |

Table S9. Association between use special products and psycho-sociodemographic characteristics, results of univariate analyses.

| Used special products such as powdered supplements  Yes (reference category is No) | | | | |
| --- | --- | --- | --- | --- |
|  | OR | 95% CI | p-value | p-value^1^ |
| Age (years)  <39 years  ≥40 years | 0.53  REF | 0.33, 0.87 | 0.01 | 0.29 |
| Gender  Male  Female | 0.78  REF | 0.47, 1.27 | 0.32 | 1.00 |
| Country of birth  Australia  Other | 1.55  REF | 0.85, 2.85 | 0.15 | 1.00 |
| Marital status  Married or defacto  Other | 0.72  REF | 0.38, 1.37 | 0.33 | 1.00 |
| Income  ≤$99,999  >$99,999 | 1.03  REF | 0.63, 1.69 | 0.87 | 1.00 |
| Smoker (yes)  No  Yes | 1.28  REF | 0.54, 3.05 | 0.56 | 1.00 |
| Weight stable in last 3 months?  No, increased  No, decreased  Yes | 0.62  0.42  REF | 0.34, 1.15  0.17, 1.01 | 0.13 | 1.00 |
|  |  |  | 0.05 | 0.90 |
| Weight acceptance  Yes  No | 0.54  REF | 0.32, 0.91 | 0.02 | 0.40 |
| Presence of comorbid conditions  No  Yes | 0.77  REF | 0.47, 1.25 | 0.30 | 1.00 |
| Weight  Baseline  Change over 12m | 0.02  0.00 | 0.00  0.02 | <0.01 | <0.01 |
|  |  |  | 0.88 | 1.00 |
| QOL - Physical health domain score  Baseline  Change over 12m | -0.15  -0.01 | 0.05  0.06 | <0.01 | <0.01 |
|  |  |  | 0.87 | 1.00 |
| QOL - Psychological domain score  Baseline  Change over 12m | -0.14  -0.01 | 0.04  0.07 | <0.01 | <0.01  1.00 |
|  |  |  | 0.88 |  |
| QOL - Social relationships domain score  Baseline  Change over 12m | -0.09  0.00 | 0.04  0.04 | 0.02 | 0.40 |
|  |  |  | 0.91 | 0.91 |
| QOL - Environment domain score  Baseline  Change over 12m | -0.07  0.01 | 0.06  0.07 | 0.21 | 1.00 |
|  |  |  | 0.85 | 1.00 |
| Depression  Baseline  Change over 12m | 0.04  -0.01 | 0.01  0.02 | <0.01 | <0.01 |
|  |  |  | 0.41 | 1.00 |
| Anxiety  Baseline  Change over 12m | 0.02  0.04 | 0.01  0.02 | 0.16 | 1.00 |
|  |  |  | 0.10 | 1.00 |
| Stress  Baseline  Change over 12m | 0.03  -0.02 | 0.01  0.02 | 0.04 | 0.76 |
|  |  |  | 0.18 | 1.00 |
| ^1^ P-values adjusted using Holm-Bonferroni correction method | | | | |

Table S10. Association between use special products and psycho-sociodemographic characteristics, results of multivariate analyses.

| Used special products such as powdered supplements  Yes (reference category is No) | | | |
| --- | --- | --- | --- |
|  | Estimate | SE | p-value |
| Intercept | -0.73 | 1.43 | 0.60 |
| Weight at baseline | 0.01 | 0.01 | <0.01 |
| QOL - Physical health domain score at baseline | -0.08 | 0.06 | 0.19 |
| QOL - Psychological domain score at baseline | -0.03 | 0.08 | 0.63 |
| Depression at baseline | 0.01 | 0.02 | 0.58 |

Table S11. Association between fasting and psycho-sociodemographic characteristics, results of univariate analyses.

| Fasted  Yes (reference category is No) | | | | |
| --- | --- | --- | --- | --- |
|  | OR | 95% CI | p-value | Adjusted p-value^1^ |
| Age (years)  <39 years  ≥40 years | 1.12  REF | 0.74, 1.70 | 0.58 | 1.00 |
| Gender  Male  Female | 0.76  REF | 0.50, 1.16 | 0.21 | 1.00 |
| Country of birth  Australia  Other | 1.12  REF | 0.68, 1.83 | 0.64 | 1.00 |
| Marital status  Married or defacto  Other | 0.67  REF | 0.37, 1.19 | 0.17 | 1.00 |
| Income  ≤$99,999  >$99,999 | 0.89  REF | 0.58, 1.37 | 0.61 | 1.00 |
| Smoker (yes)  No  Yes | 1.18  REF | 0.57, 2.42 | 0.64 | 1.00 |
| Weight stable in last 3 months?  No, increased  No, decreased  Yes | 0.44  0.35  REF | 0.25, 0.77  0.15, 0.85 | <0.01 | <0.01 |
|  |  |  | 0.02 | 0.42 |
| Weight acceptance  Yes  No | 0.49  REF | 0.32, 0.77 | <0.01 | <0.01 |
| Presence of comorbid conditions  No  Yes | 0.96  REF | 0.63, 1.46 | 0.85 | 1.00 |
| Weight  Baseline  Change over 12m | 0.00  -0.02 | 0.00  0.0 | 0.24 | 1.00 |
|  |  |  | 0.30 | 1.00 |
| QOL - Physical health domain score  Baseline  Change over 12m | -0.10  -0.02 | 0.04  0.06 | 0.02 | 0.42 |
|  |  |  | 0.72 | 1.00 |
| QOL - Psychological domain score  Baseline  Change over 12m | -0.17  0.05 | 0.04  0.06 | <0.01 | <0.01 |
|  |  |  | 0.36 | 1.00 |
| QOL - Social relationships domain score  Baseline  Change over 12m | -0.07  0.00 | 0.03  0.04 | 0.03 | 0.54 |
|  |  |  | 0.93 | 0.93 |
| QOL - Environment domain score  Baseline  Change over 12m | -0.19  0.08 | 0.05  0.06 | <0.01 | <0.01 |
|  |  |  | 0.25 | 1.00 |
| Depression  Baseline  Change over 12m | 0.03  0.00 | 0.01  0.01 | 0.03 | 0.54 |
|  |  |  | 0.82 | 1.00 |
| Anxiety  Baseline  Change over 12m | 0.03  0.00 | 0.01  0.02 | 0.03 | 0.54 |
|  |  |  | 0.93 | 0.93 |
| Stress  Baseline  Change over 12m | 0.02  0.00 | 0.01  0.01 | 0.12 | 1.00 |
|  |  |  | 0.88 | 1.00 |
| ^1^ P-values adjusted using Holm-Bonferroni correction method | | | | |

Table S12. Association between fasting and psycho-sociodemographic characteristics, results of multivariate analyses.

| Fasted  Yes (reference category is No) | | | |
| --- | --- | --- | --- |
|  | Estimate | SE | p-value |
| Intercept | 3.24 | 1.06 | <0.01 |
| QOL - Psychological domain score at baseline | -0.10 | 0.05 | 0.05 |
| QOL - Environment domain score at baseline | -0.06 | 0.07 | 0.39 |
|  | OR | 95% CI | p-value |
| Weight stable in last 3 months?  No, increased  No, decreased  Yes | 0.33  0.62  REF | 0.12, 0.87  0.21, 1.84 | 0.02  0.39 |
| Weight acceptance  Yes  No | 0.65  REF | 0.40, 1.07 | 0.09 |

Table S13. Association between self-vomiting and psycho-sociodemographic characteristics, results of univariate analyses.

| Caused self to vomit after eating  Yes (reference category is No) | | | | |
| --- | --- | --- | --- | --- |
|  | OR | 95% CI | p-value | Adjusted p-value^1^ |
| Age (years)  <39 years  ≥40 years | 1.49  REF | 0.24, 9.03 | 0.66 | 1.00 |
| Gender  Male  Female | 0.87  REF | 0.14, 5.29 | 0.88 | 0.88 |
| Country of birth  Australia  Other | 0.20  REF | 0.03, 1.24 | 0.08 | 1.00 |
| Marital status  Married or defacto  Other | NA | NA |  |  |
| Income  ≤$99,999  >$99,999 | 0.36  REF | 0.04, 3.32 | 0.37 | 1.00 |
| Smoker (yes)  No  Yes | 0.41  REF | 0.04, 3.80 | 0.43 | 1.00 |
| Weight stable in last 3 months?  No (increase or decrease)  Yes | 0.45  REF | 0.07, 2.74 | 0.38 | 1.00 |
| Weight acceptance  Yes  No | 2.38  REF | 0.39, 14.47 | 0.34 | 1.00 |
| Presence of comorbid conditions  No  Yes | 1.88  REF | 0.31, 11.39 | 0.49 | 1.00 |
| Weight  Baseline  Change over 12m | -0.04  -0.09 | 0.03  0.08 | 0.20 | 1.00 |
|  |  |  | 0.25 | 1.00 |
| QOL - Physical health domain score  Baseline  Change over 12m | -0.30  0.28 | 0.15  0.20 | 0.04 | 0.84 |
|  |  |  | 0.16 | 1.00 |
| QOL - Psychological domain score  Baseline  Change over 12m | -0.21  -0.23 | 0.15  0.21 | 0.16 | 1.00 |
|  |  |  | 0.29 | 1.00 |
| QOL - Social relationships domain score  Baseline  Change over 12m | 0.09  -0.05 | 0.15  0.16 | 0.55 | 1.00 |
|  |  |  | 0.72 | 1.00 |
| QOL - Environment domain score  Baseline  Change over 12m | -0.44  0.39 | 0.21  0.26 | 0.04 | 0.84 |
|  |  |  | 0.13 | 1.00 |
| Depression  Baseline  Change over 12m | 0.06  0.07 | 0.04  0.06 | 0.15 | 1.00 |
|  |  |  | 0.30 | 1.00 |
| Anxiety  Baseline  Change over 12m | 0.11  -0.03 | 0.04  0.08 | 0.01 | 0.24 |
|  |  |  | 0.66 | 1.00 |
| Stress  Baseline  Change over 12m | 0.08  -0.07 | 0.04  0.05 | 0.04 | 0.84 |
|  |  |  | 0.15 | 1.00 |
| ^1^ P-values adjusted using Holm-Bonferroni correction method | | | | |

Table S14. Association between use of any other weight change strategies and psycho-sociodemographic characteristics, results of univariate analyses.

| Anything else to lose or maintain weight  Yes (reference category is No) | | | | |
| --- | --- | --- | --- | --- |
|  | OR | 95% CI | p-value | Adjusted p-value^1^ |
| Age (years)  <39 years  ≥40 years | 0.87  REF | 0.57, 1.33 | 0.53 | 1.00 |
| Gender  Male  Female | 0.41  REF | 0.26, 0.65 | <0.01 | <0.01 |
| Country of birth  Australia  Other | 1.91  REF | 1.11, 3.27 | 0.01 | 0.23 |
| Marital status  Married or defacto  Other | 0.87  REF | 0.48, 1.58 | 0.66 | 1.00 |
| Income  ≤$99,999  >$99,999 | 1.01  REF | 0.65, 1.56 | 0.94 | 1.00 |
| Smoker (yes)  No  Yes | 1.42  REF | 0.66, 3.07 | 0.36 | 1.00 |
| Weight stable in last 3 months?  No, increased  No, decreased  Yes | 0.50  0.42  REF | 0.29, 0.88  0.18, 0.99 | 0.01 | 0.23 |
|  |  |  | 0.04 | 0.84 |
| Weight acceptance  Yes  No | 0.60  REF | 0.38, 0.94 | 0.02 | 0.44 |
| Presence of comorbid conditions  No  Yes | 0.34  REF | 0.21, 0.53 | <0.01 | <0.01 |
| Weight  Baseline  Change over 12m | 0.00  -0.02 | 0.00  0.02 | 0.24 | 1.00 |
|  |  |  | 0.38 | 1.00 |
| QOL - Physical health domain score  Baseline  Change over 12m | -0.08  -0.02 | 0.04  0.06 | 0.05 | 1.00 |
|  |  |  | 0.64 | 1.00 |
| QOL - Psychological domain score  Baseline  Change over 12m | -0.04  0.03 | 0.04  0.06 | 0.34 | 1.00 |
|  |  |  | 0.59 | 1.00 |
| QOL - Social relationships domain score  Baseline  Change over 12m | 0.05  -0.03 | 0.03  0.04 | 0.11 | 1.00 |
|  |  |  | 0.38 | 1.00 |
| QOL - Environment domain score  Baseline  Change over 12m | 0.00  0.02 | 0.05  0.07 | 0.95 | 1.00 |
|  |  |  | 0.75 | 1.00 |
| Depression  Baseline  Change over 12m | 0.00  0.00 | 0.01  0.01 | 0.95 | 1.00 |
|  |  |  | 0.99 | 0.99 |
| Anxiety  Baseline  Change over 12m | 0.02  0.01 | 0.01  0.02 | 0.15 | 1.00 |
|  |  |  | 0.58 | 1.00 |
| Stress  Baseline  Change over 12m | 0.02  -0.01 | 0.01  0.01 | 0.12 | 1.00 |
|  |  |  | 0.35 | 1.00 |
| ^1^P-values adjusted using Holm-Bonferroni correction method | | | | |

Table S15. Association between use of any other weight change strategies and psycho-sociodemographic characteristics, results of multivariate analyses.

| + | | | |
| --- | --- | --- | --- |
|  | Estimate | SE | p-value |
| Intercept | 0.15 | 0.16 | 0.35 |
|  | OR | 95% CI | p-value |
| Gender  Male  Female | 0.43  REF | 0.27, 0.69 | <0.01 |
| Presence of comorbid conditions  No  Yes | 0.35  REF | 0.22, 0.56 | <0.01 |
